# Mass resection as a candidate treatment for uterine PEComas of uncertain malignant potential: a case report and literature review

**DOI:** 10.3389/fonc.2024.1521253

**Published:** 2025-01-27

**Authors:** Haijuan Shi, Yan Yin, Shoujun Liang, Chuanzhong Liu, Yibao Huang, Bingfeng Lu, Liying Zhang

**Affiliations:** ^1^ Department of Gynaecology, The Second Affiliated Hospital of Guangxi Medical University, Nanning, China; ^2^ Department of Radiology, The Second Affiliated Hospital of Guangxi Medical University, Nanning, China

**Keywords:** uterine PEComas, perivascular epithelioid cell, uncertain malignant potential, fertility preservation, case report

## Abstract

**Background:**

Perivascular epithelioid cell tumours (PEComas) occurring in the uterus are rare, with surgery being the most recommended primary treatment for malignant cases. This study aims to provide clinical guidance on the clinicopathological features and appropriate treatment options for patients with uterine PEComas of uncertain malignant potential.

**Cases:**

This case series summarises the clinical courses of 13 patients diagnosed with uterine PEComas of uncertain malignant potential, including clinical and pathological data as well as their outcomes. We identified one case at our hospital, and data for the other 12 cases were extracted from the PubMed database. The 13 patients were aged 9–75 years, with tumour sizes ranging from 1 to 21 cm, and follow-up times ranging from 2 to 71 months. The most common signs and symptoms included abnormal uterine bleeding (AUB) and abdominal pain. Most of the patients (11/13) were managed surgically without any chemotherapy or radiation therapy. Except for the patients who were lost to follow-up, 11 patients were free of any recurrence or metastasis at their last follow-up. Patients with group A tumours (abundant HMB45 expression) had a longer disease-free survival than those with group B tumours.

**Conclusions:**

Surgery alone may be appropriate for uterine PEComas of uncertain malignant potential. Surgical treatment plans should consider the patient’s age, fertility requirements, and personal preferences. Mass resection is a viable treatment option for fertility preservation in reproductive-age patients.

## Introduction

Perivascular epithelioid cell tumours (PEComas) are very rare mesenchymal tumours characterised by the expression of both melanocytic and myogenic markers (1). The World Health Organisation (WHO) previously defined PEComas as mesenchymal tumours composed of histologically and immunohistochemically distinctive perivascular epithelioid cells (2). PEComas are known to occur at various anatomical locations, including the lung, kidney, bladder, and prostate. Nearly a quarter of PEComas occur in the female genital tract (3), and uterine PEComas were described initially by Bonetti (4).

The clinical behaviour of uterine PEComas is usually benign; these cases display benign features and show no evidence of recurrence or metastasis after surgery alone (5). However, the malignant potential of uterine PEComas is variable, and a proportion of cases may recur and metastasise. Bennett suggested that all gynaecological PEComas should be classified as either malignant or of uncertain malignant potential (6). In such cases, complete surgical resection and chemotherapy are crucial for preventing clinical recurrence (7). Therefore, PEComas with uncertain malignant potential present great difficulties in medical decision-making. However, the criteria for PEComas of uncertain malignant potential have not been established by the WHO due to their rarity. Based on the criteria proposed by Folpe et al. (8), a PEComa of “uncertain malignant potential” is defined as having only a single high-risk histological feature, such as nuclear pleomorphism or multinucleated giant cells, or a size > 5 cm. Owing to the paucity of cases, there are controversies over the management of uterine PEComas of uncertain malignant potential, especially in reproductive-age patients. This study summarises the clinical course of uterine PEComas of uncertain malignant potential and explores the possibility of preserving fertility in these patients.

## Case presentation

A 35-year-old woman was referred to our hospital presented with a 3-day history of lower abdominal pain. Ultrasound revealed a solid mass measuring 10.9 cm × 9.3 cm × 9.3 cm on the left side of the uterus (**Supplementary Figure S1A**). Pelvic magnetic resonance imaging (MRI) later showed a solid mass in the left pelvis, measuring 10.5 cm × 8.2 cm × 9.5 cm with uneven enhancement (**Supplementary Figures S1B, C**). A metastatic workup, including whole-body computed tomography (CT) and serum tumour marker detection, was negative for disease. Therefore, subserosal uterine fibroids with degeneration were considered, although ovarian cyst torsion could not be ruled out. After obtaining informed consent, a mass resection was performed. Macroscopically, the mass was located in the left broad ligament, with a maximum diameter of 11 cm. It appeared dark purple, had an uneven surface, and displayed a beef-like soft texture. A malignant tumour was not considered based on intraoperative frozen pathology. To maintain the patient’s fertility, no additional operations were performed. Histological analysis revealed that the tumour consisted of round and spindle cells with an eosinophilic granular cytoplasm. The tumour cells presented slight to moderate nuclear atypia, with nuclear enlargement and distinct nucleolus. No mitotic figures, vascular invasion, haemorrhage, or necrosis were observed (**Supplementary Figure S2A**). The immunohistochemical results were as follows: positive staining for SMA (**Supplementary Figure S2B**), desmin (**Supplementary Figure S2C**), melan A (**Supplementary Figure S2D**), Ki-67 (20%), and TFE3 (**Supplementary Figure S2E**). Negative staining was observed for HMB45, CK(Pan), S-100, CD117, CD34, MyoD1, SDHB, H-caldesmon, and SOX-10. In addition, fluorescence *in situ* hybridisation (FISH) did not detect *TFE3* gene rearrangement (**Supplementary Figure S2F**). A uterine PEComa of uncertain malignant potential was diagnosed based on the above findings. The patient has been undergoing regular physical examinations and remained free of disease at 8 months after surgery.

## Review of the literature

**Figure 1:**
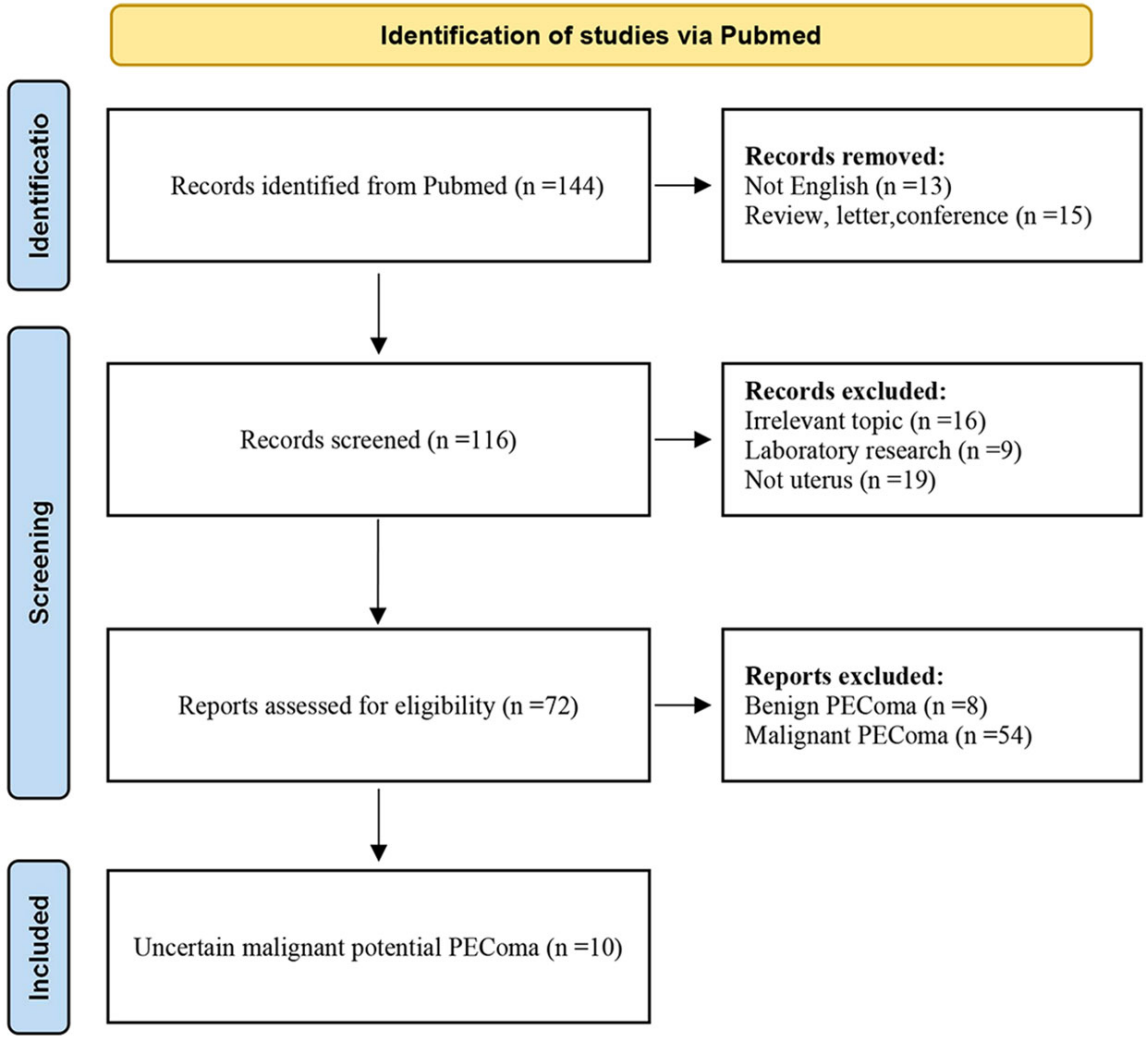
A flowchart showing the selection process for the 10 selected studies.

Previous cases were extracted from the PubMed database and analysed to provide additional information. The final search was conducted in May 2024. Two researchers independently screened eligible publications based on titles and abstracts using the following search query: (perivascular epithelioid cell neoplasm) OR (perivascular epithelioid cell tumours) OR (PEComa*). All English-language articles reporting uterine PEComas of uncertain malignant potential with available full texts were included. The selection flowchart is presented in **Figure 1**. As of 2024, 12 cases were extracted from the PubMed database (5, 9–17). The clinicopathological features of the patients, including their clinical manifestations, pathology, treatment, and outcomes, are summarised in **Table 1**. The 13 patients were aged 9–75 years (mean: 36.5 years), with tumour sizes ranging from 1 to 21 cm (mean: 8.5 cm) and follow-up durations ranging from 2 to 71 months (mean: 17.5 months). The most common signs and symptoms included abnormal uterine bleeding (AUB) and abdominal pain.

**Table 1:** Clinicopathologic features of patients with uterine PEComa of uncertain malignant potential.

Case	Country	Age (year)	Initial presentation	Tumor size (cm)	Preoperative diagnosis	High-risk feature	Treatment	Follow-up (months)
Case 1	China	29	Abdominal pain	12	Leiomyoma	Tumor size>5cm	Mass resection	8
Case 2 (9)	India	29	AUB	21	Uterine sarcoma	Tumor size>5cm	Hysterectomy	12
Case 3 (10)	Hispanic	39	Abdominal pain	8.5	Uterine PEComa	Tumor size>5cm	Hysterectomy	4
Case 4 (11)	China	23	Abdominal pain	10	Ovarian cyst torsion	Tumor size>5cm	Mass resection	71
Case 5 (12)	China	44	AUB	7	Unknown	Tumor size>5cm	Hysterectomy	2
Case 6 (13)	Thailand	38	Abdominal pain	7	Leiomyoma	Tumor size>5cm	Mass resection	7
Case 7 (14)	Japan	34	Infertility	5	Leiomyoma	Tumor size>5cm	Mass resection	12
Case 8 (14)	Japan	51	None	7	Ovarian tumor	Tumor size>5cm	Hysterectomy	24
Case 9 (15)	Denmark	38	AUB	1	Leiomyoma	Nuclear pleomorphism	Hysterectomy	10
Case 10 (16)	Pakistan	25	AUB	3.5	Adenocarcinoma	Nuclear pleomorphism	Hysterectomy	UK
Case 11 (17)	Korean	9	Abdominal pain	6.5	Uterine PEComa	Tumor size>5cm	Hysterectomy, chemotherapy, radiation therapy	18
Case 12 (5)	America	40	Hemoperitoneum	12	UK	Tumor size>5cm	Mass resection	UK
Case 13 (5)	America	75	AUB	10	UK	Tumor size>5cm	Hysterectomy, radiation therapy	30

AUB, abnormal uterine bleeding; UK, unknown.

**Figure 2:**
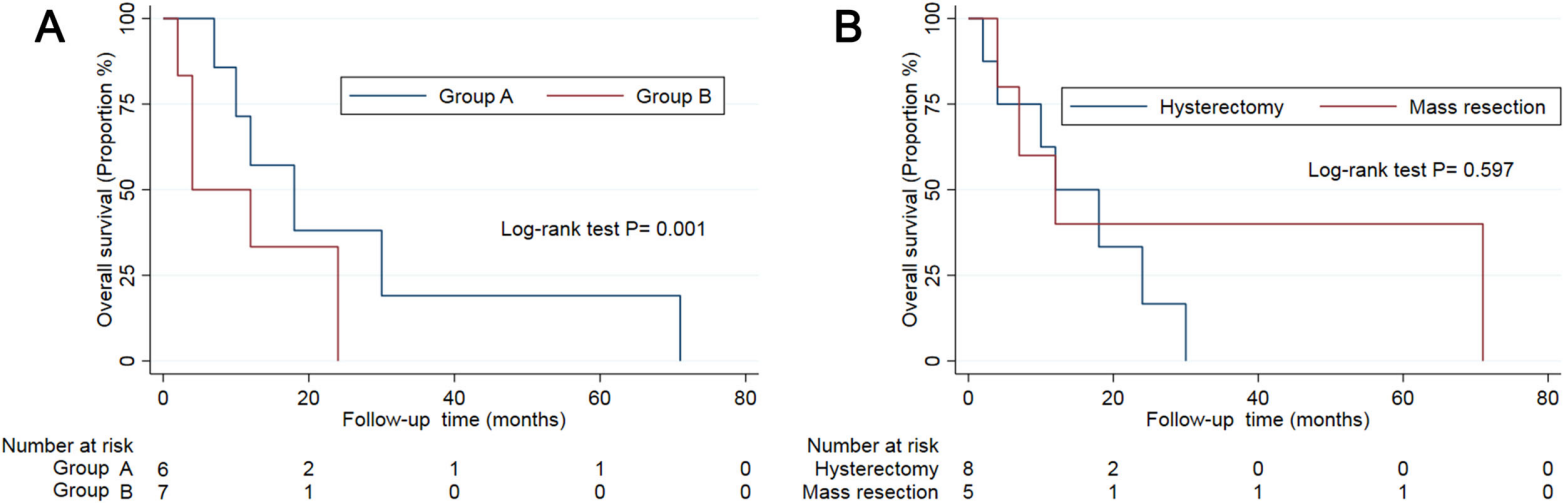
The survival curve of patients in subgroups by immunohistochemistry **(A)** and treatment **(B)**.

The immunohistochemical profiles of the 13 patients are presented in **Supplementary Table S1**. We further separated these patients into two groups based on the immunohistochemical expression as follows: group A tumours typically exhibited high HMB-45 expression but low muscle marker expression, whereas group B tumours exhibited the opposite pattern (5). In this study, six patients had group A tumours. The mean age of the patients was 33 years. Three patients presented with AUB, while others presented with abdominal pain. Four patients underwent hysterectomy, with one of them receiving chemotherapy and radiation therapy (17). The other two patients were treated via mass resection. Clinical follow-up data were available for five patients, with follow-up times ranging from 7 to 71 months (mean: 27.6 months). Conversely, seven patients had group B tumours, with a mean age of 39 years. These patients exhibited diverse symptoms: two presented with AUB, two with abdominal pain, one with haemoperitoneum, and two reported no apparent discomfort. Four patients underwent hysterectomy, while the remaining patients underwent mass resection. Clinical follow-up data were available for six patients, with durations ranging from 2 to 24 months (mean: 10 months) (**Table 2**). Excluding patients lost to follow-up, all 11 remaining patients were free of recurrence or metastasis at their last follow-up. Patients with group A tumours demonstrated longer disease-free survival compared to those with group B tumours (**Figure 2A**).

**Table 2:** Clinical features of patients with uterine PEComas of uncertain malignant potential, categorised by subgroups.

Variable	Total	Immunohistochemistry group		*p*-value	Treatment		*p*-value
		Group A (*n* = 6)	Group B (*n* = 7)		Mass resection (*n* = 5)	Hysterectomy (*n* = 8)	
Age (years)	36.5 ± 4.4	33.2 ± 22.6	39.3 ± 7.0	0.75	32.8 ± 6.9	38.8 ± 19.5	0.27
Tumour size (cm)	8.5 ± 1.4	9.7 ± 6.1	7.5 ± 3.9	0.23	9.2 ± 3.1	8.1 ± 5.9	0.65
Follow-up time (months)	17.5 ± 5.0	27.6 ± 25.7	10.0 ± 7.8	0.07	23.0 ± 27.1	14.6 ± 9.6	0.78

To date, therapeutic protocols for uterine PEComas of uncertain malignant potential have not been established, with treatment based on protocols for managing malignant PEComas. In this study, all 13 patients underwent surgical excision, including eight who underwent hysterectomy and five who underwent mass resection. Most of the patients (11/13) were treated surgically without receiving chemotherapy or radiation therapy. Disease-free survival did not differ significantly between patients who underwent hysterectomy and those who underwent mass resection for uterine PEComas of uncertain malignant potential (**Table 2**, **Figure 2B**). Due to the paucity of cases, the role of chemotherapy and radiation therapy remains unclear.

## Discussion

PEComas are rare mesenchymal tumours that can occur in various anatomical locations. Nearly a quarter of PEComas are found in the female genital tract, most frequently arising in the uterus. In this study, we report a case of uterine PEComas in a 35-year-old woman who presented with sudden lower abdominal pain but no other specific symptoms. Ultrasound and MRI imaging revealed a palpable mass, though typical presentations were absent. Consequently, the serum tumour markers in this patient were negative. Therefore, the nonspecific symptoms and imaging findings posed challenges in making a preoperative diagnosis of uterine PEComas.

In addition, defining the behaviour and prognosis of uterine PEComas is challenging due to their rarity. Folpe et al. (8) proposed risk stratification criteria based on five high-risk histopathological features: a large tumour size of more than 5 cm, high nuclear grade and cellularity, a mitotic rate more than 1/50 HPF, an infiltrative growth pattern, and the presence of necrosis and vascular invasion. In particular, uterine PEComas of uncertain malignant potential had a tumour size larger than 5 cm or exhibited only nuclear pleomorphism or multinucleated giant cells. The differential diagnosis of uterine PEComas included leiomyoma, uterine sarcoma, ovarian tumour, and adenocarcinoma (**Table 1**). Notably, the uterine PEComas in the 13 patients shared clinical features similar to those of leiomyoma, including an abdominal mass, AUB, and abdominal pain. Only two cases were diagnosed with uterine PEComas before surgery. Therefore, uterine PEComas of uncertain malignant potential are often misdiagnosed as uterine leiomyoma. A distinctive feature is that melanocytic markers are positive in PEComas but negative in leiomyomas.

All 13 patients were diagnosed after surgery through pathology and immunohistochemicals. In most cases, the uterine PEComas had exhibited characteristic immunohistochemical features, including immunoreactivity for HMB45 and negativity for the S-100 protein. However, case 1 was negative for HMB45 expression (**Supplementary Table S1**). Based on the immunohistochemical expression of HMB45 and muscle markers, we separated uterine PEComas into two groups. Group A tumours typically presented abundant HMB45 expression but scant muscle marker expression and appeared to be associated with longer disease-free survival than did the group B tumours. Due to the variable malignant potential of PEComas, 50% of gynaecological PEComas are considered to have malignant potential (18). Although the optimal management of uterine PEComas is still controversial, surgical excision remains the preferred treatment, followed by adjuvant chemotherapy and radiotherapy (1). Our study revealed that surgery alone appeared to be appropriate for uterine PEComas of uncertain malignant potential, particularly for those with a tumour size > 5 cm.

However, the diagnoses were made based on pathological reports combined with immunohistochemical staining, which has inevitable hysteresis. Therefore, uterine PEComas of uncertain malignant potential present significant challenges in making decision-making during a second operation, especially for patients who have undergone only mass resection. Shan et al. suggested that mass resection is sufficient if the uterine PEComas tend are pathologically benign, but long-term follow-up is still necessary (11). As a typical example, case 4 involved a 23-year-old unmarried woman diagnosed with uterine PEComas. Given the consideration for fertility preservation, she underwent laparoscopic mass resection and continued with long-term follow-up. The patient subsequently became pregnant spontaneously and gave birth to a healthy boy via caesarean section 5 years after the surgery. Moreover, she had the longest disease-free survival period among the 13 cases. Therefore, the patient’s age, fertility requirements, and personal preferences should be considered when developing surgical treatment plans.

## Strengths and limitations

This study represents the largest case series of uterine PEComas with uncertain malignant potential, including one case from our institution and 12 additional cases from a literature database. Although the case series is small, this limitation is inherent to any rare condition. In addition, some patients only had short-term follow-ups or were even lost to follow-up, highlighting the importance of long-term monitoring to gain a deeper understanding of this condition.

## Conclusions

PEComas occurring in the uterus are very rare. Surgery alone may be suitable for uterine PEComas with uncertain malignant potential. Surgical treatment plans should consider the patient’s age, fertility requirements, and personal preferences. Mass resection is a potential treatment option for fertility preservation in reproductive-age patients.

## Data Availability

The datasets presented in this study can be found in online repositories. The names of the repository/repositories and accession number(s) can be found in the article/**Supplementary Material**.

## Supplementary material

Supplementary Figure 1 Transvaginal ultrasound (A) and Magnetic resonance imaging (BC) of case 1

Supplementary Figure 2 Histopathologic view of uterine PEComas of Case 1. **(A)** Hematoxylin-eosin staining, **(B)** Immunohistochemical staining for SMA. **(C)** Immunohistochemical staining for Desmin. **(D)** Immunohistochemical staining for melan A. **(E)** Immunohistochemical staining for TFE3. **(F)** Fluorescence *in situ* hybridization for TFE3. (**Supplementary Figure S2D**), Ki-67 (20%) and (**Supplementary Figure S2E**).
