# Supplementary figures and images for: Mass resection as a candidate treatment for uterine PEComas of uncertain malignant potential: a case report and literature review
Source: Front Oncol. 2025 Jan 27;14:1521253. doi: 10.3389/fonc.2024.1521253 (PMC11807954; doi:10.3389/fonc.2024.1521253)

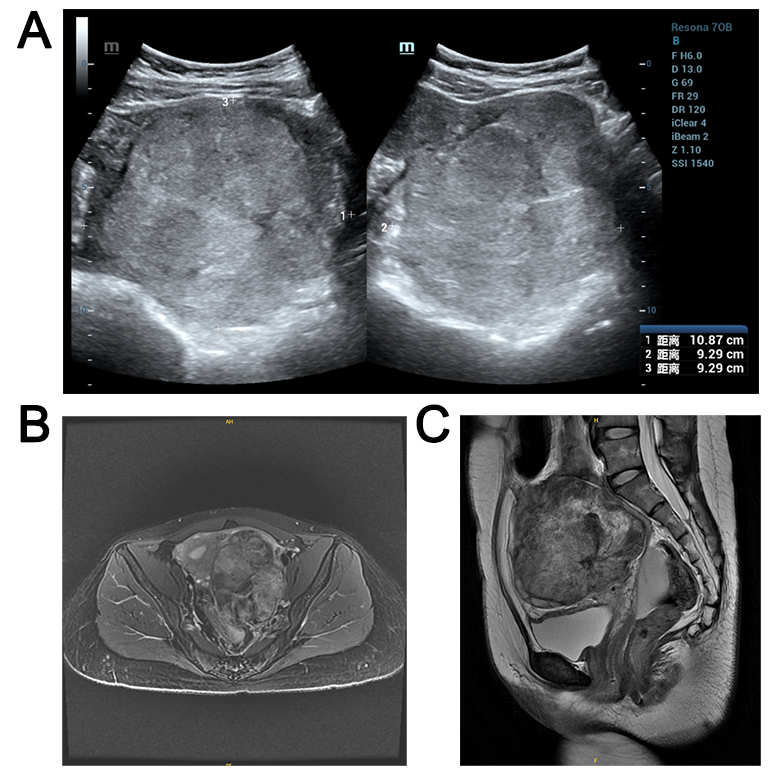

Supplement: Supplementary Figure 1 — Transvaginal ultrasound (A) and Magnetic resonance imaging (BC) of case 1 [file Image1.tif]

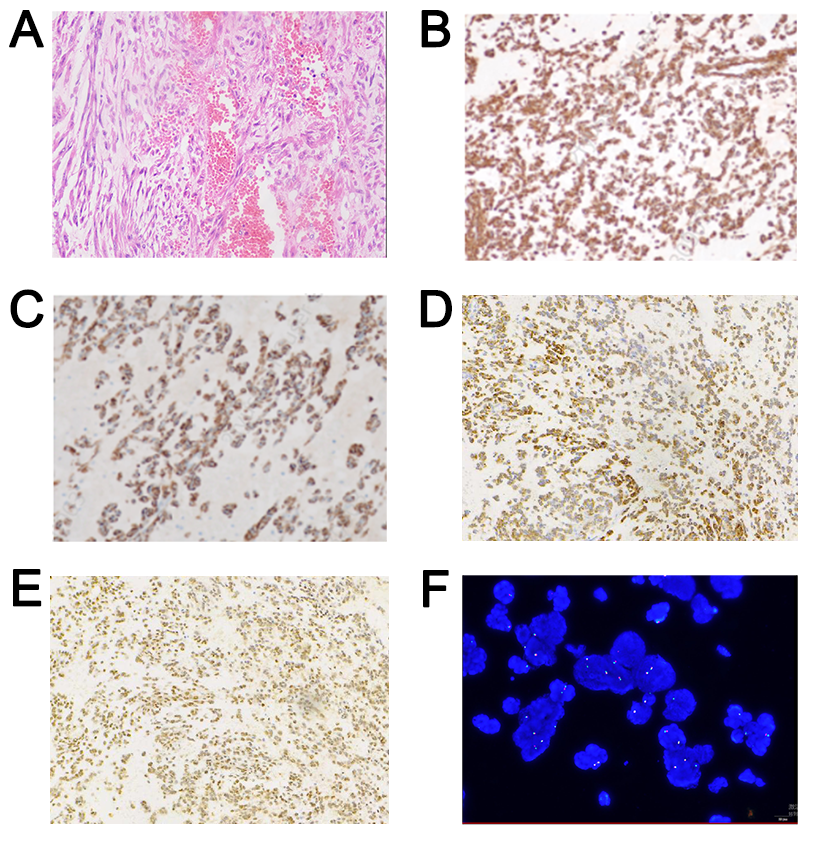

Supplement: Supplementary Figure 2 — Histopathologic view of uterine PEComas of Case 1. (A) Hematoxylin-eosin staining, (B) Immunohistochemical staining for SMA. (C) Immunohistochemical staining for Desmin. (D) Immunohistochemical staining for melan A. (E) Immunohistochemical staining for TFE3. (F) Fluorescence in situ hybridization for TFE3. ( Supplementary Figure S2D ), Ki-67 (20%) and ( Supplementary Figure S2E ). [file Image2.tif]
