# Supplementary material for: Mass resection as a candidate treatment for uterine PEComas of uncertain malignant potential: a case report and literature review
Source: Front Oncol. 2025 Jan 27;14:1521253. doi: 10.3389/fonc.2024.1521253 (PMC11807954; doi:10.3389/fonc.2024.1521253)
Supplement: Supplementary file 3 [file Table1.docx]

Table S1. Immunohistochemical profile of patients with uterine PEComa of uncertain malignant potential.

| Case | Immunohistochemical profile | | | | | | | Group |
| --- | --- | --- | --- | --- | --- | --- | --- | --- |
|  | HMB-45 | SMA | Melan-A | Desmin | Vimentin | S-100 | Cytokeratin |  |
| Case 1 | - | + | + | + | UK | - | - | B |
| Case 2 | + | - | UK | + | + | UK | - | A |
| Case 3 | + | + | UK | UK | UK | UK | UK | B |
| Case 4 | + | UK | UK | UK | UK | UK | UK | A |
| Case 5 | + | + | + | + | + | - | UK | B |
| Case 6 | + | UK | - | - | UK | - | UK | A |
| Case 7 | + | + | - | + | + | - | - | B |
| Case 8 | + | + | - | + | + | - | - | B |
| Case 9 | + | + | UK | - | UK | UK | UK | B |
| Case 10 | + | - | - | - | + | - | - | A |
| Case 11 | + | - | - | - | - | - | - | A |
| Case 12 | + | + | + | UK | UK | - | - | B |
| Case 13 | + | - | - | UK | UK | - | - | A |

Note: SMA, smooth muscle actin; UK, unknown.
